# Alternative splicing of a group II intron in a surface layer protein gene in *Clostridium tetani*

**DOI:** 10.1093/nar/gkt1053

**Published:** 2013-11-08

**Authors:** Bonnie A. McNeil, Dawn M. Simon, Steven Zimmerly

**Affiliations:** Department of Biological Sciences, University of Calgary, 2500 University Dr. NW, Calgary, Alberta T2N 1N4, Canada

## Abstract

Group II introns are ribozymes and retroelements found in bacteria, and are thought to have been the ancestors of nuclear pre-mRNA introns. Whereas nuclear introns undergo prolific alternative splicing in some species, group II introns are not known to carry out equivalent reactions. Here we report a group II intron in the human pathogen *Clostridium tetani*, which undergoes four alternative splicing reactions *in vivo*. Together with unspliced transcript, five mRNAs are produced, each encoding a distinct surface layer protein isoform. Correct fusion of exon reading frames requires a shifted 5′ splice site located 8 nt upstream of the canonical boundary motif. The shifted junction is accomplished by an altered IBS1-EBS1 pairing between the intron and 5′ exon. Growth of *C. tetani* under a variety of conditions did not result in large changes in alternative splicing levels, raising the possibility that alternative splicing is constitutive. This work demonstrates a novel type of gene organization and regulation in bacteria, and provides an additional parallel between group II and nuclear pre-mRNA introns.

## INTRODUCTION

Group II introns are large catalytic RNAs and retroelements found within prokaryotic genomes, as well as in organellar genomes of eukaryotes such as fungi, plants and protists (1–3). The introns possess a conserved secondary structure of six domains, which folds into a catalytically active structure capable of self-splicing (4–6). The ribozymes typically self-splice utilizing a two-step transesterification mechanism to produce ligated exons and lariat intron, although a minority of group II introns splice by a hydrolysis pathway to form linear excised intron instead (7,8).

In bacteria, most group II introns encode an open reading frame (ORF) within the loop of domain 4 that allows the introns to be mobile and insert into new genomic locations, sometimes with high efficiency (9–11). The intron-encoded protein (IEP) is a multi-domain protein that contains a reverse transcriptase (RT), a maturase (X), a DNA binding (D) and an optional endonuclease (En) domain. Despite the self-splicing capability of group II introns, splicing *in vivo* requires the IEP to stabilize the catalytically active RNA structure. The IEP also functions in the integration of the intron sequence into new genomic locations through well-studied retrohoming and retrotransposition mechanisms (12–15). This mobile form of group II introns, consisting of both ribozyme and RT-related IEP, predominates in bacteria and is considered the ancestral form of extant group II introns (16,17).

A number of striking similarities exist between group II introns and nuclear pre-mRNA introns, which have led to the hypothesis that group II introns were the predecessors of nuclear pre-mRNA introns and the spliceosome (18,19). The similarities include identical chemical splicing pathways and the formation of RNA pairings during spliceosome assembly that are analogous to group II RNA structures (20). Recently, the relationship between group II and spliceosomal introns was strongly supported by the X-ray crystal structure of the spliceosomal protein Prp8. The structure depicts a RT-derived domain located in the heart of the spliceosome, within direct cross-linking distance to the intron and exon substrates and to the snRNAs (21). The structure is consistent with the scenario that the spliceosome derived from a mobile group II intron, and contains remnants of both a self-splicing ribozyme and a RT IEP.

A hallmark of spliceosomal introns not shared by group II introns is the ability to alternatively splice. Eukaryotic introns do this through mechanisms of intron retention, exon skipping and alternate 5′ and 3′ splice-site selection, thereby increasing transcriptome and proteome complexity (22). For example, ∼95% of human pre-mRNAs are alternatively spliced (23), and in an extreme example the *D. melanogaster dscam* gene can theoretically generate 38 016 protein isoforms through alternative splicing (24).

For group II introns, equivalent alternative splicing has not been reported, although there are two reports of related reactions. In *Euglena gracilis* chloroplasts, several group II introns can splice using alternative 5′ or 3′ splice sites (25,26). However, these reactions occur at low frequencies and produce ORF truncations or small insertions, and as such may reflect aberrant processing rather than beneficial alternative reactions. In a second example, the *B.a*.I2 intron of *Bacillus anthracis* utilizes two 3′ splice sites located 4 nt apart. Splicing with the upstream site produces protein from the 5′ exon ORF only, while splicing with the downstream site (∼4% of mRNA products) correctly fuses the upstream and downstream ORFs to produce a two domain protein (27). The biological functions of the ORFs are unknown. Despite the similarities, neither the *Euglena* chloroplast nor the *B. anthracis* reaction fully resembles eukaryotic-like alternative splicing in ligating together different reading frames to produce multiple protein isoforms.

The present study concerns a group II intron in a surface layer protein (SLP) gene. Surface layers (S-layers) are crystalline-like, two-dimensional arrays of proteinaceous subunits on the outermost surface of most eubacteria and virtually all archaebacteria (28). They are typically composed of a single primary protein or glycoprotein, which self-assembles into the S-layer through contacts between crystallization domains. In addition to providing a barrier to the external environment, S-layers in Gram-positive bacteria are reported to affect virulence, provide resistance to the host immune response and play roles in cellular adhesion (29).

The composition of S-layers and the amino acid sequences of SLPs are highly variable, both across and within species. For example, three strains of *Geobacillus stearothermophilus* each express a distinct SLP, and two of the strains express an alternate SLP under conditions of oxygen or elevated temperatures (30,31). Interestingly, the oxygen-induced SLP switch is due to a DNA rearrangement between chromosomal and plasmid loci that encode the two SLP genes (30). DNA inversions have similarly been shown to control SLP or cell wall gene expression in *Campylobacter* and *Clostridium difficile* (32,33). Thus, many mechanisms are used by different bacteria to generate variability in S-layer and cell wall proteins.

Here we report the bioinformatic identification and experimental characterization of *C.te.*I1, a novel group II intron in the bacterial pathogen *Clostridium tetani*. We show that the intron undergoes four alternative splicing reactions *in vivo* to produce mRNAs encoding five SLP isoforms. In addition to demonstrating a novel gene organization in bacteria, the work underscores the evolutionary adaptability of group II introns and increases functional similarities between group II and nuclear pre-mRNA introns.

## MATERIALS AND METHODS

### Strains and growth conditions

*Clostridium tetani* strain ATCC10779 (Designation 43415—Harvard Strain; American Type Culture Collection) is closely related to the sequenced strain E88. Over the course of this study, all sequence in the ∼6.5 kb region of *C.te.*I1 was PCR amplified and sequenced, and confirmed to be identical to strain E88, with the exception of a single gap of 559 bp that was not amplified (intergenic region between CTC00468 and CTC00469). *Clostridium tetani* cultures were grown in Brain Heart Infusion Medium (Oxoid CM1135) at 37°C under anaerobic conditions using the GasPak EZ anaerobic container system (BD Biosciences). For stress conditions, cells were grown under anaerobic conditions to mid-logarithmic phase before the introduction of the stressor (see Supplementary Materials).

### RNA and DNA preparations

RNA extraction was performed using the RNeasy Mini Kit (Qiagen). Cells were harvested and lysed according to the Appendix C protocol of the RNAprotect Bacteria Reagent Handbook (Second Edition, December 2005). Protocol 7 was used for the purification of either total RNA or DNA from bacterial lysate, either with or without optional on-column DNase digestion. Integrity of RNA preparations were visualized on a 1.2% agarose gel and purity and concentration were assessed by OD_260_/OD_280_.

### Plasmid constructs

The *C.te*.I1 intron was PCR amplified from *C. tetani* genomic DNA in two separate pieces using the primers S-5SSC-Bam, AS-5SSC-Eco, S-3SSC-Eco and AS-3SSC-Cla (Supplementary Table S1). The two pieces were ligated by recombinant PCR, and cloned into the BamHI and ClaI sites of pBluescript KS+ (Stratagene). The resulting plasmid (pWT-SSC) contained 91 nt of the 5′ exon (CTC00465) and 80 nt of the 3′ exon (CTC00467), and was deleted for 315 bp of loop sequence in domain 4. Site-directed mutagenesis of EBS1 and IBS1 was performed as previously described (34) using 5′ phosphorylated oligos and Pfu DNA polymerase.

### *In vitro* transcription and self-splicing assays

Transcription reactions were performed at 37°C for 30 min in a volume of 20 µl of 40 mM Tris-HCl (pH 8.0), 4 mM MgCl_2_, 50 mM NaCl, 1 mM each NTP, 5 mM DTT, 0.05% Triton X-100, and with 500 ng plasmid template (linearized with XhoI) and T7 RNA polymerase. The reaction was extracted with phenol-chloroform isoamyl alcohol (25:24:1) and precipitated with ethanol and 2.5 M NH_4_OAc. For radiolabelled transcripts, reactions contained 1 µl of [α-^32^P] UTP (10 mCi/ml, 3000 Ci/mmol, MP Biomedicals). For self-splicing reactions, ^32^P-labelled (100000 cpm) or cold transcript (200 ng) was re-suspended in TE and folded by the following incubations: 90°C for 1 min, 75°C for 5 min and slow cooling to 45°C over 15 min. Self-splicing buffer was added to produce a volume of 50 μl containing 100 mM MgCl_2_, 0.5 M NH_4_Cl and 40 mM Tris-HCl (pH 7.5). Reactions were incubated at 53°C for 5 min, followed by ethanol precipitation and resolution on a 4% polyacrylamide (19:1 acrylamide:bisacrylamide ratio)/8 M urea gel. For the unspliced control, reactions were done in parallel with the omission of MgCl_2_.

### RT-PCR and qRT-PCR

cDNA synthesis was performed in 20 μl using 10 pmole gene-specific primer (O1R, I1R2, O2R4, O3R3, O4R2 or O5R3; Supplementary Table S2) and 200 U Superscript II RT (Invitrogen) with 1 µg total RNA as template, according to manufacturer’s protocol. No-RT controls were performed with the omission of RT. qPCR reactions were in a total volume of 12.5 µl containing 10 pmol of forward (O1F, O1F4) and reverse primers (O1R, I1R2, O2R4, O3R3, O4R2, O5R3), 6.25 µl 2X iQ SYBR Green Supermix (Bio-Rad) and 2 μl of the previous RT reaction as template. A three-cycle amplification was performed (95°C for 10 s, 57°C for 20 s, 72°C for 20 s) with melt curve (ramping from 72°C to 95°C) using the Rotor-Gene Q, real-time PCR cycler (Qiagen). No-RT and no-template controls were included in each run, and all unknowns were run in technical triplicates. Standard curves were made from serial dilutions of plasmids with cloned exon junctions of identical sequence to allow for quantification of unknown samples. All primer pairs amplified with 95–105% efficiency. Total RNA samples obtained were assessed for quality, integrity and purity prior to qRT-PCR. For non-quantitative RT-PCR, PCR reactions were performed as above with Pfu DNA polymerase in 10 mM Tris-HCl (pH8.8), 2.5 mM MgCl_2_, 50 mM KCl and 0.1% Triton X-100.

## RESULTS

### Bioinformatic identification of *C.te.*I1

**Figure 1.**
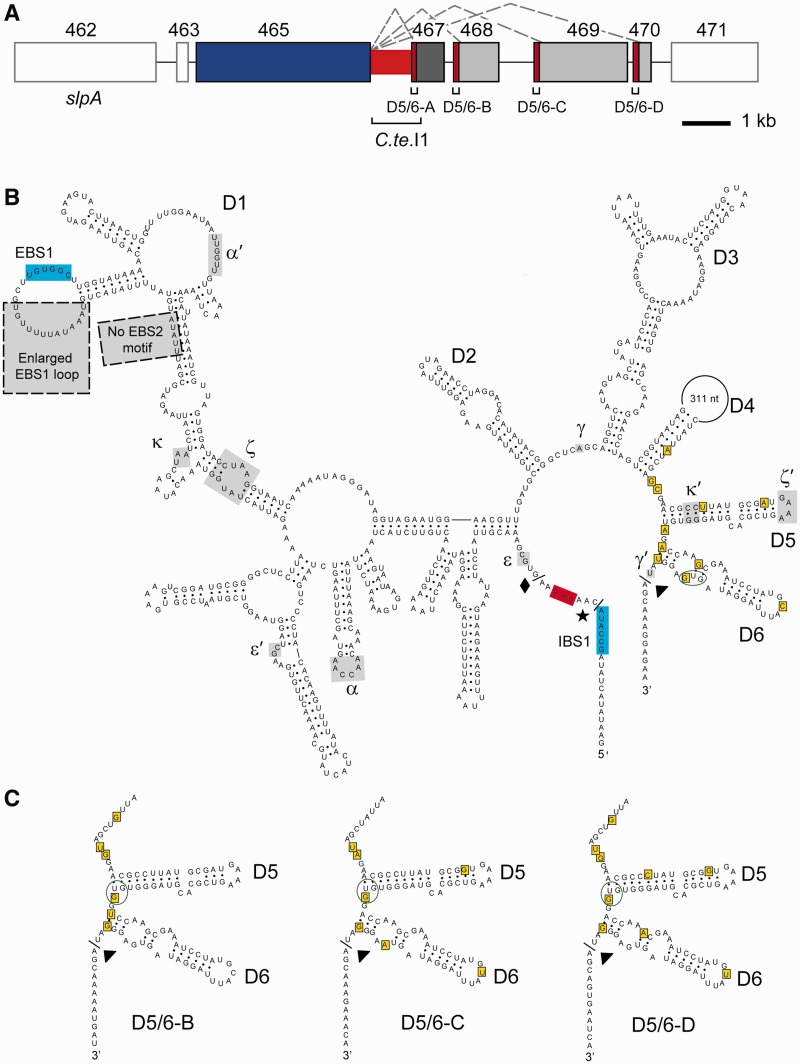
Genomic arrangement and the intron secondary structure. (**A**) The *C.te*.I1 intron (red) is located between annotated ORFs CTC00465 (blue) and CTC00467 (dark grey), and is downstream of the major SLP gene of *C. tetani* (*slpA*, CTC00462). Three downstream D5/6 motifs (outlined red boxes; D5/6-B, C, D) are followed by ORFs CTC00468, CTC00469 and CTC00470 (medium grey), together forming a series of four alternative 3′ splice sites. Dotted grey lines indicate potential alternative splicing reactions, and thin black lines are intergenic sequences. The diagram is drawn to scale. ORF numbers 462–471 refer to annotated ORFs CTC00462–CTC00471. (**B**) The secondary structure model of *C.te.*I1 is typical of Class B, but has significant structural variations near the EBS1 motif (grey boxes with dotted outline). The sequence of the 311 nt loop in domain 4 is not shown. The 5′ splice site predicted by the group II boundary motif is indicated with a black diamond, while the actual 5′ splice site is shown with a black star and the 3′ intron boundary by a black triangle. The UAA stop codon of ORF CTC00465 is located between the predicted and actual 5′ splice sites, and is indicated with red shading. The IBS1-EBS1 pairing sequences are labelled with light blue shading, and other predicted tertiary interactions are indicated by Greek letters and grey shading. Yellow-boxed nucleotides in domains 4b, 5 and 6 indicate polymorphisms among the four D5/6 motifs. The annotated start codon for the downstream exon is circled in green. (**C**) Secondary structures of the downstream domain 5 and 6 motifs, highlighting the sequence polymorphisms (yellow boxes) and annotated start codons (green circles) (compare with Panel B).

During a bioinformatic search for ORF-less group II introns in bacterial genomes (17), a novel intron-related sequence was identified in the genome of *C. tetani* E88. Within a 5.1 kb region, four intron domain 5 (D5) motifs were detected, each of nearly identical sequence, and each followed by a domain 6 (D6) motif (Figure 1). For the upstream D5/6 motif, an entire intron could be elucidated by secondary structure modelling, which we call *C.te*.I1 (Figure 1B); however, the RNA structure possesses several unconventional features in domain I, including an expansion of the EBS1 loop and the absence of an EBS2 motif, along with its corresponding IBS2–EBS2 pairing. In sequence and structure, *C.te.*I1 is most similar to introns of Class B, which is one of eight phylogenetic clades of mobile group II introns in bacteria. Introns in this class are abundant in Firmicutes and account for over half of the known group II introns in *Clostridium* and *Bacillus* genera [(17); and unpublished data]. Unlike the majority of bacterial group II introns, *C.te*.I1 does not encode an IEP to aid splicing, nor is there an IEP-related sequence in the remainder of the sequenced genome that might act in *trans*. In addition, the 311 nt loop in domain 4 does not bear sequence similarity to group II IEPs or any proteins in GenBank.

*C.te.*I1 lies on the *C. tetani* chromosome (CTC) in a ∼75 kb region containing a large array of SLP genes. At the beginning of this region is the *slpA* gene (CTC00462), which is the predominant SLP expressed in *C. tetani* (35). Approximately 4 kb downstream of *slpA* and immediately downstream of an annotated SLP (CTC00465) is *C.te*.I1. All four D5/6 motifs in the locus are followed by annotated ORFs (CTC00467–CTC00470), whose start codons are in the D5/6 motifs (Figure 1A–C). The downstream ORFs share sequence similarity to cell wall proteins, transglutaminases and/or proteases (see below and Table 1).

**Table 1.** Inferred functions of exon-encoded proteins

Annotated ORF	Exon location	GenBank annotation^a^	BLASTP matches^b^	Pfam matches^c^
CTC00465	5′ exon	Putative S-layer protein	Hypothetical protein,	No matches
			Cell wall-binding protein,	
			Extracellular nuclease	
CTC00467	3′ exon	Hypothetical protein	Hypothetical protein	No significant matches
CTC00468	3′ exon	Predicted transglutaminase/protease	Transglutaminase/protease,	Transglutaminase-like superfamily
			Cell wall-binding protease,	
			S-layer protein	
CTC00469	3′ exon	Predicted transglutaminase/protease	Cell wall-binding protease,	Transglutaminase-like superfamily
			Transglutaminase/protease,	
			S-layer protein	
CTC00470	3′ exon	Hypothetical protein	S-layer protein,	No significant matches
			Transglutaminase/protease,	
			Hypothetical protein	

^a^Protein function listed in GenBank annotation.

^b^The three most common functional descriptions of protein matches.

(e < 2e-07; except for CTC00470 where matches were between 4.4 and 0.008) (www.ncbi.nlm.nih.gov/BLAST/).

^c^Protein family domains identified by Pfam 3.0 (www.pfam.sanger.ac.uk).

Based on the predicted secondary structure, splicing of *C.te.*I1 would join CTC00465 and CTC00467 into one mRNA; however, the ORFs would not be fused because the 5′ boundary of the intron (5′GUGCG) lies 2 nt downstream of the stop codon of CTC00465 (Figure 1B). In addition, splicing would remove the annotated start codon of CTC00467 in D5/6, and presumably disrupt translation of the downstream ORF. Potential alternative splicing reactions involving domains 1–4a of *C.te*.I1 and any of the three downstream D5/6 motifs would similarly fail to link the reading frames and would remove the start codons of the downstream ORFs (CTC00468–CTC00470). Overall, the functionality of *C.te*.I1 was initially unclear due to the unusual secondary structure features, the absence of an IEP and the predicted failure to ligate the exon reading frames.

### *C.te*.I1 splices *in vivo* to produce five distinct coding sequences

**Figure 2.**
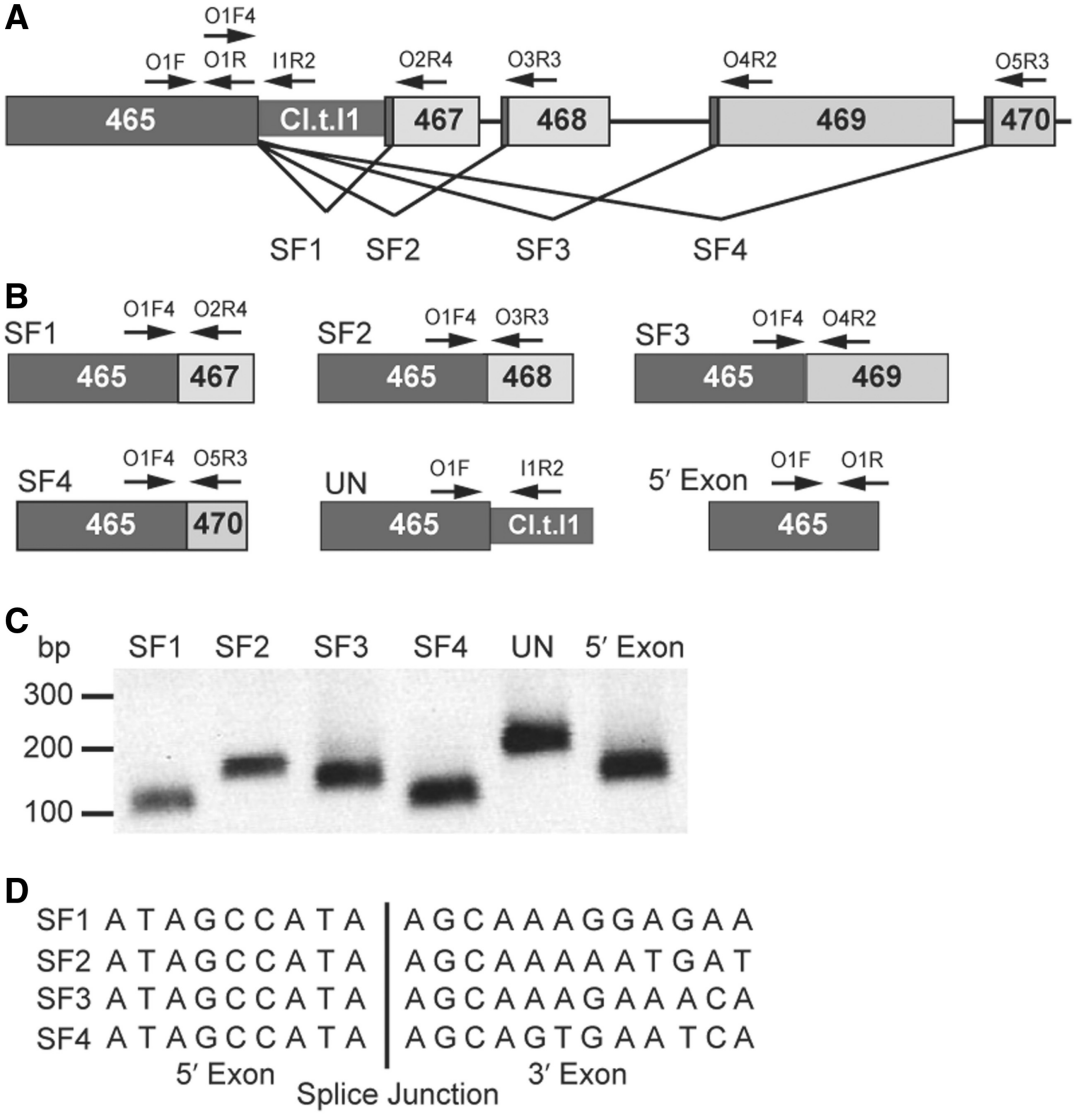
Alternative splicing *in vivo*. (**A**) The four alternative splicing reactions are indicated by black lines joining CTC00465 with downstream exon ORFs (splicing forms SF1–4). Primers for PCR reactions are represented by arrows. (**B**) Diagram of spliced exons and the RNAs amplified by RT-PCR. (**C**) Agarose gel of RT-PCR amplification products. RNA preparations were digested with DNase I prior to RT-PCR reactions, and RT-PCR products were dependent on RT (data not shown). (**D**) Sequences of cloned splice junctions. ORF numbers 465–470 refer to annotated ORFs CTC00465–CTC00470.

Splicing of *C.te*.I1 was analysed in the *C. tetani* strain ATCC10779, a close relative of the sequenced Massachusetts substrain E88. Both strains are derivatives of the Harvard strain that overproduces the tetanus toxin and has been used in vaccine production (36). Total cellular RNA from *C. tetani* was assayed by RT-PCR for potential splicing reactions. DNAs were amplified with all combinations of primers to detect the four predicted splicing products, as well as the unspliced transcript and 5′ exon (Figure 2). DNA sequencing confirmed the four splicing reactions but also revealed that the splice junctions did not conform to the predicted intron boundaries: for each of the spliced products, the 5′ intron boundary was shifted 8 nt upstream of the conserved 5′ boundary motif (5′GUGCG, Figure 1B). Importantly, the use of this novel splice site eliminates the stop codon of the upstream ORF (CTC00465) and results in the in-frame ligation of the flanking exons (i.e., CTC00465–CTC00467, CTC00465–CTC00468, CTC00465–CTC00469 and CTC00465–CTC00470). Therefore, *C.te.*I1 is functional for splicing *in vivo*, and is capable of four alternative splicing reactions, which along with unspliced transcript produces five distinct coding mRNAs.

### The novel 5′ splice site is specified by the ribozyme activity of *C.te*.I1

**Figure 3.**
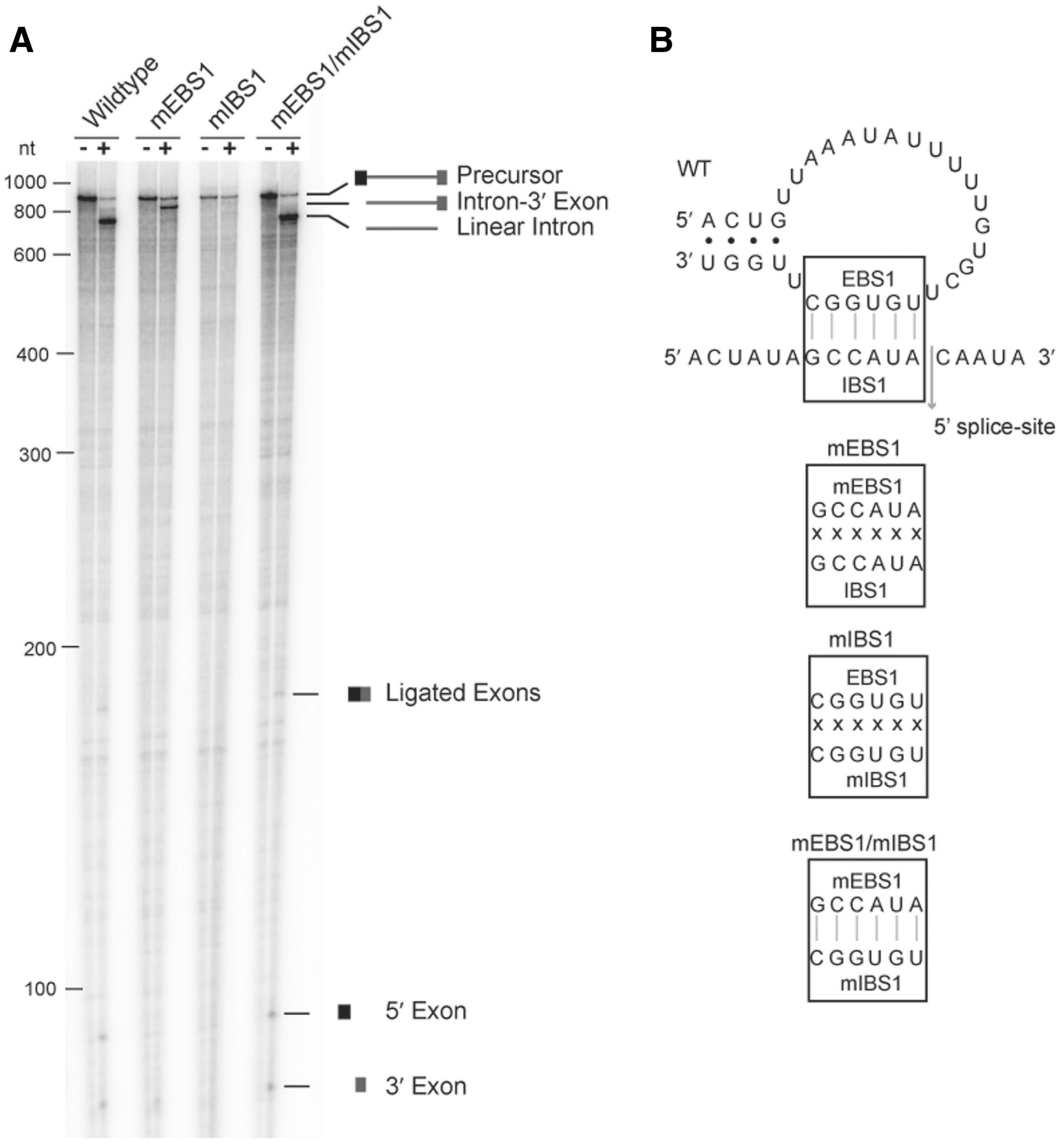
Usage of the novel 5′ splice site is due to the ribozyme properties of *C.te.*I1. (**A**) Self-splicing reactions of wild-type *C.te.*I1 transcript (WT), transcripts with mutant EBS1 (mEBS1) or IBS1 (mIBS1) sequences, or compensatory mutations in both EBS1 and IBS1 sequences (mEBS1/mIBS1). Minus and plus signs indicate unspliced precursor and products, respectively, after a 10 min splicing reaction. (**B**) The EBS1 and IBS1 mutations tested in Panel A.

The novel 5′ splice site could be specified by either the intrinsic ribozyme property of *C.te*.I1, or by splicing factors present in the cell. To address this issue, *C.te*.I1 was assayed for self-splicing *in vitro*. RNA transcripts were found to self-splice efficiently, albeit through a hydrolysis pathway, to produce ligated exons and linear intron (Figure 3). The ligated exons were amplified by RT-PCR and sequenced, which revealed the same shifted 5′ splice site observed for *in vivo* splicing. We conclude that the novel 5′ splice site is due to the intrinsic catalytic reaction of the ribozyme rather than by auxiliary protein splicing factors present *in vivo*.

For group II introns, 5′ exons are positioned by two pairing interactions, exon-binding sites 1 and 2 (EBS1 and 2), located in domain 1, and intron-binding sites 1 and 2 (IBS1 and 2), located immediately upstream of the splice site (37). An EBS2 motif is not evident in the secondary structure model of *C.te.*I1, whereas a potential EBS1 sequence is present in a loop at the distal end of domain 1. The putative EBS1 sequence lies in a 24 nt loop that is dramatically larger than the typical EBS1 loop size of 9–10 nt. To test whether the candidate EBS1 and IBS1 sequences pair with each other and are responsible for 5′ splice selection, the sequences were mutated alone and in combination (Figure 3). Consistent with our hypothesis, mutation of either sequence blocked self-splicing while the compensatory mutations restored splicing, thus demonstrating a bona fide pairing interaction between the two sequences, which is required for the splice sites observed *in vivo*. Overall, the self-splicing data clearly show that the ribozyme is robustly catalytic despite its structural deviations from the predicted ancestral mobile intron form. The maintenance of ribozyme activity strongly argues against the possibility that the *C.te.*I1 locus is a non-functional genomic remnant of a previously active intron.

### Quantification of alternatively spliced RNAs *in vivo*

In eukaryotes, most well-studied examples of alternative splicing are highly regulated, such that specific protein isoforms are produced in various tissue types, at various developmental stages or in response to external stimuli. However, alternative splicing can also occur constitutively, in which a constant ratio of isoforms is generated from a single gene. It has been noted that the true frequency of constitutive alternative splicing is unknown and may be underreported (22).

**Figure 4.**
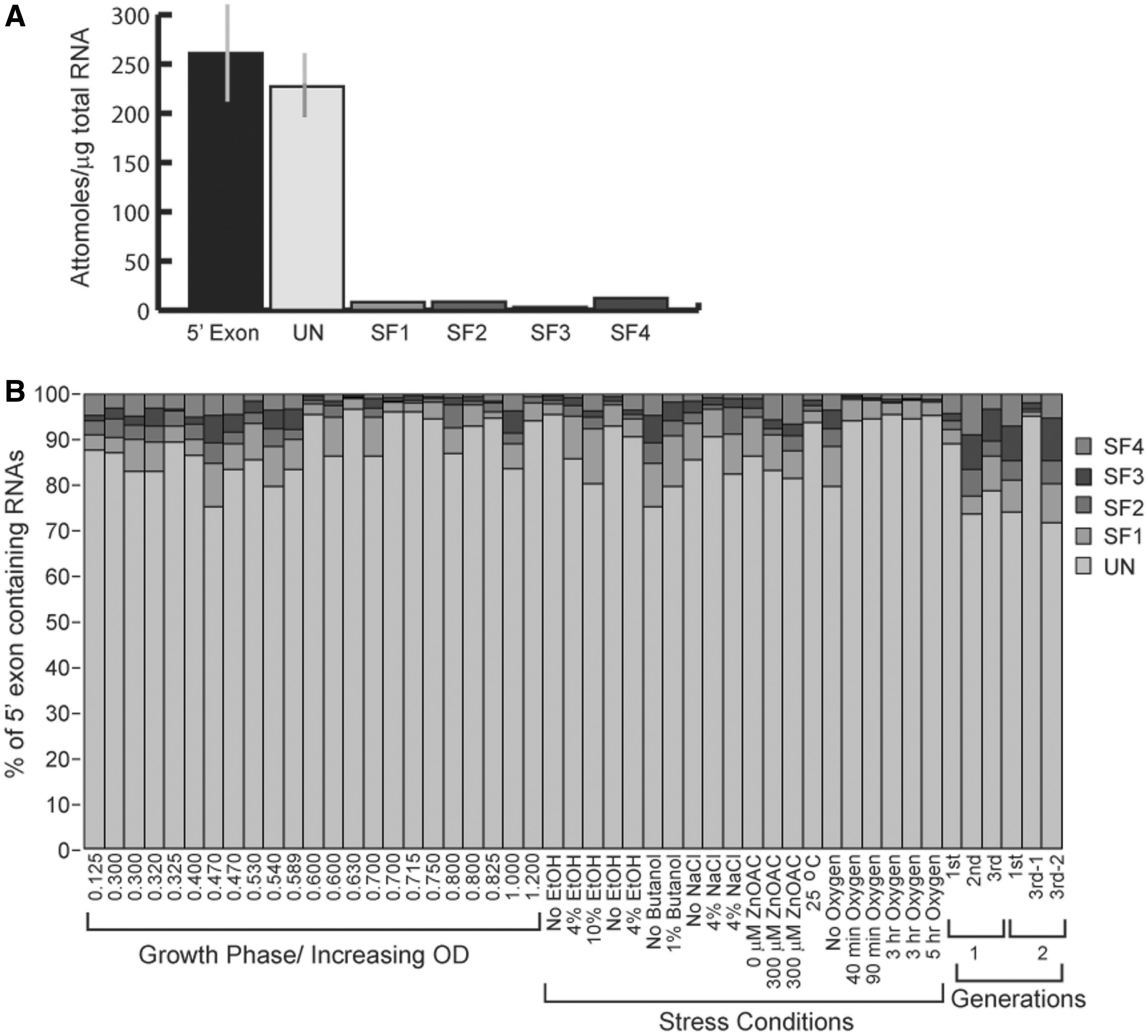
Quantification of splice forms by qRT-PCR. (**A**) Quantification of RNAs present in 1 μg of total RNA isolated from *C. tetani* cultures grown to an OD_600_ of 0.125. The four splice forms, unspliced RNA and 5′ exon were quantified, and molar amounts were calculated based on calibrations with DNA standards of identical sequence and known quantity (see ‘Materials and Methods’ section). (**B**) qPCR quantification of RNA forms present for various growth conditions. Values are expressed as % of the total molar amounts of the five RNAs. For a detailed description of conditions, see Supplementary Materials. A full table of values with standard deviations is in Supplementary Table S1.

To address whether alternative splicing of *C.te*.I1 is constitutive or regulated, quantitative real-time reverse-transcriptase PCR (qRT-PCR) experiments were performed. Six RNA segments were amplified from total RNA isolated from *C. tetani* ATTC10779 cells: the four spliced exon junctions, the unspliced exon–intron junction and a 5′ exon segment (Figure 2B). In order to accurately compare amounts of the five different mRNAs produced, it was necessary to quantify absolute amounts of RNAs using standards of known quantities of cloned DNAs having identical sequence. The measurements revealed that the most predominant mRNA form present is unspliced transcript, while the other four forms are present at significantly lower but detectable levels (Figure 4A; Supplementary Table S3).

To investigate whether splicing products vary in response to growth conditions, RNA was extracted from *C. tetani* cultures subjected to stress conditions, including some of those known to influence gene expression of virulence factors in pathogenic bacteria (38). Treatments included stresses of osmotic strength, temperature, oxygen exposure and metal ions (see Supplementary Materials). *Clostridium tetani* cells were also collected throughout growth phases and over multiple experimental preparations, as well as different concentrations of the nutrient broth. Interestingly, unspliced RNA accounted for the majority of transcripts under all conditions tested, comprising ∼75–95% of mRNAs (Figure 4B). At most, modest variation was observed under any condition; however, similar variations existed as well between replicate trials of the conditions, suggesting a degree of stochastic variation rather than specifically regulated differences.

## DISCUSSION

The *C.te.*I1 intron is the first example of alternative splicing in bacteria that resembles eukaryotic alternative splicing in producing a variety of mRNAs from a single locus. To our knowledge, it is also the first example of an ORF-less group II intron in a bacterium that splices in the absence of an IEP encoded elsewhere in the genome. It seems likely that other introns similar to *C.te.*I1 exist in bacteria but have been overlooked, because group II introns are usually identified through the IEP, because many introns are located outside of conserved genes, and because there are many fragmented group II intron copies in genomes.

Group II introns are known to be evolutionarily versatile. They exist in nature in a range of forms that vary in mobility and splicing capabilities, including many forms that are highly degenerate for either the intron RNA structure (e.g., group III introns) or the IEP (1–4). *C.te.*I1 represents a new evolutionary outcome for group II introns, in which mobility function was lost, but host fitness was putatively increased through a modified mechanism of gene expression that creates protein diversity.

The biological role of alternative splicing remains enigmatic, because specific functions for the five exon-encoded ORFs are not known. Experimentally, the CTC00465 protein has been identified as a minor constituent in SLP preparations from *C. tetani* strain CN655, while three other clinical isolates did not show the protein at detectable levels (35). Thus CTC00465 is a protein component of the S-layer, but appears not to be an essential component, at least in all strains. Of the four downstream exon ORFs that become appended to CTC00465 by the alternative splicing reactions, three show similarities to transglutaminases or proteases (Table 1). Transglutaminases are a large family of enzymes that catalyse post-translational modifications via transamidation of glutamine residues, resulting in the formation of ε-(γ-glutamyl) lysine crosslinks (39), which generally increase resistance to proteolytic degradation. Protease factors are also known to be required for assembly of some S-layers. For example, SlpA in *C. difficile* is proteolytically processed into high- and low-molecular weight forms (40). In considering the biological function of alternative splicing of *C.te.*I1, it is plausible that the putative cross-linking and/or protease activities encoded by downstream exons alter the S-layer properties, either directly or through the assembly process.

Attempts to identify regulation of the splice forms under different growth conditions were unsuccessful, raising the possibility that alternative splicing occurs constitutively to produce a low level of four variant SLPs in addition to the predominant isoform of CTC00465. On the other hand, as environmental conditions are difficult to reproduce in the laboratory, it is possible that regulation occurs in response to conditions not tested, such as during infection. A third possibility is that distinct splicing forms are produced in individual cells within a population, similar to phase variation in the S-layer of *C. difficile*, in which ∼5% of cells in a population express the cell wall protein CwpV due to a DNA inversion in the promoter region (32).

Although the precise mechanism of alternative splicing is not addressed in this study, there are multiple possible scenarios. Two key questions related to the mechanism are: (i) whether the splicing reactions occur in *cis* or in *trans*; and (ii) whether there are host-encoded splicing factors that control the four splicing reactions.

Alternative splicing is generally assumed to occur in *cis*; however, there are eukaryotic examples of alternative splicing that occur in *trans* (22). For example, the *modifier of mdg4* gene *(mod(mdg4))* in *D. melanogaster* produces 28 mRNAs through a reaction in which a common 5′ exon is spliced to 1 of 28 variable 3′ exons. At least seven of the reactions occur in *trans* (41)*.* A *trans-*splicing mechanism for *C.te*.I1 is a definite possibility, because group II introns are well known to *trans-*splice in organellar genomes (42–46). In nearly all cases of organellar *trans*-splicing, the intron fragmentation point is in domain 4, as would be the case for *C.te*.I1 if it *trans*-spliced. It is therefore possible that the 5′ and 3′ exons are transcribed separately and *trans*-spliced together, rather than being part of a single precursor transcript. An implication of a *trans*-splicing mechanism is that the four splicing reactions could be regulated at the transcriptional level rather than through splicing factors. There are as yet no examples of natural bacterial *trans-*splicing introns, although *trans-*splicing has been demonstrated to occur for certain genetic constructs in *Escherichia coli* (47,48).

In most cases, splicing of group II introns *in vivo* requires an IEP and/or host splicing factor(s) to enable the ribozyme to achieve its catalytic conformation under physiological conditions. The absence of an IEP in the *C. tetani* genome suggests either that no protein splicing factors are required for *C.te*.I1’s reactions, or more likely that other unidentified splicing factors are encoded in the genome. For example, there may be a separate protein factor to promote and regulate each of the four splicing reactions.

**Figure 5.**
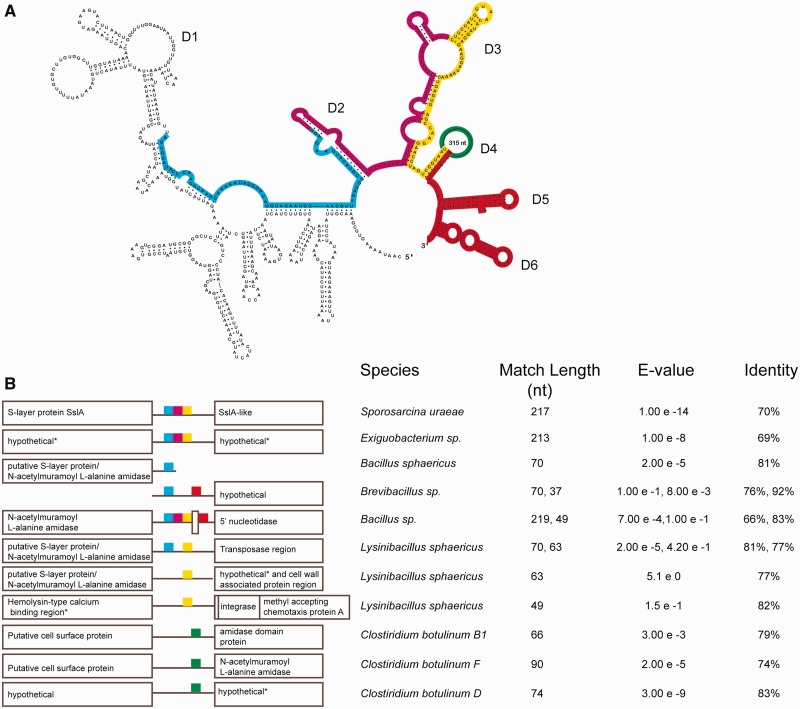
Ribozyme-derived sequences among SLP genes in various Firmicutes. (**A**) Secondary structure of *C.te.I1*, highlighting the regions corresponding to BLASTN matches in other species. (**B**) Coloured boxes indicate BLASTN matches between the corresponding region of *C.te.I1* in Panel A and the indicated genomic sequence (matches are not shown to scale). Hits corresponding to D5/6 were included only if hits of D1–4 were also observed. The match length, E-value and % identity for each high-scoring pair (HSP) is shown to the right. Accession numbers: *Sporosarcina uraeae* ATCC13881, AM293285.1; *Exiguobacterium* sp. AT1b, CP001615.1; *Bacillus sphaericus* 2363 (partial genome), M28361.1; *Brevibacillus* sp. BC25 (WGS contig), AKIX010000101.1; *Bacillus* sp. B14905 (WGS contig), AAXV01000043.1; *Lysinibacillus sphaericus* C3-41, CP000817.1; *C. botulinum* B1 strain okra, CP000939.1; *C. botulinum* F strain 230613, CP002011.1; *C. botulinum* F strain langeland, CP000728.1; *C. botulinum* D strain 1873, ACSJ01000007.1.

The *C.te*.I1 alternative splicing arrangement appears to have arisen relatively recently, as it is not found in any other sequenced *Clostridium* genomes. In fact, strain E88 is the only *C. tetani* genome sequence available, and so it is unknown how widespread the *C.te*.I1 locus is across *C. tetani* strains. BLAST searches of GenBank do not reveal any closely related introns, although intriguingly there are many apparent intron remnants found among S-layer genes in diverse Gram-positive species (Figure 5). BLASTN searches using *C.te.*I1 sequence as the query identified best hits corresponding to ribozyme domains 1–4 of *C.te*.I1, located adjacent to annotated cell surface and amidase genes. We speculate that the *C.te.*I1 locus was formed by an ancestral mobile intron of Class B, which had a homing site in a surface-layer-encoding region of the genome. In most bacteria where this occurred (e.g., the examples in Figure 5), the introns became inactivated and lost, whereas intron insertions in *C. tetani* led to a complex arrangement capable of alternative splicing.

Interestingly, the ribozyme of the *C.te*.I1 intron appears to have evolved along a similar pathway as a subgroup of mitochondrial IIB1 introns located within rRNA genes (49). The rRNA introns also utilize shifted 5′ splice sites, splice through hydrolysis and have a number of secondary structure features analogous to *C.te*.I1, such as loss of the EBS2 motif. Common to both *C.te*.I1 and the IIB1 mitochondrial introns is the loss of retromobility, which may be a factor in their similar structural and functional evolution (49). Because the ribozyme structure of *C.te*.I1 is not the focus of this manuscript, the parallels of the two intron types will be discussed in greater depth in another manuscript (McNeil and Zimmerly, submitted).

Finally, we note that the properties of *C.te*.I1 suggest that alternative splicing could have occurred prior to genesis of the spliceosome. The origin of alternative splicing is not certain; in some cases, alternatively spliced introns appear to have arisen recently, e.g., in the primate lineage (50), whereas other alternative splicing organizations are inferred to be ancient, dating back to the unicellular ancestor of plants, animals and fungi (51). In the eukaryotic ancestor where the spliceosome emerged, it has been hypothesized that the genome was replete with group II introns, and that group II introns were a driving force in the development of eukaryotic cells (19,52,53). Such an abundance of group II introns in a genome would provide ample opportunities for the formation of alternative splicing organizations such as *C.te*.I1. We suggest that *C.te*.I1 provides an example for how alternative splicing might have functioned before the emergence of the spliceosome and nuclear pre-mRNA introns.

## SUPPLEMENTARY DATA

Supplementary Data are available at NAR Online.

## FUNDING

Canadian Institutes of Health Research (CIHR) [MOP-93662 to S.Z.]; PGS-D studentship from the Natural Sciences and Engineering Research Council, Canada (to B.A.M.); Alberta Ingenuity fellowship (to D.M.S.). Funding for open access charge: CIHR.

*Conflict of interest statement*. None declared.
